# 非小细胞肺癌驱动基因研究进展

**DOI:** 10.3779/j.issn.1009-3419.2015.01.07

**Published:** 2015-01-20

**Authors:** 建国 赵, 建萍 熊

**Affiliations:** 1 312000 绍兴，绍兴市人民医院/浙江大学绍兴医院肿瘤内科 Department of Medical Oncology, Shaoxing People's Hospital, Shaoxing Hospital of Zhejiang University, Shaoxing 312000, China; 2 330006 南昌，南昌大学第一附属医院肿瘤内科 Department of Medical Oncology, the First Affiliated Hospital of Nanchang University, Nanchang 330006, China

**Keywords:** 肺肿瘤, 腺癌, 鳞癌, 癌基因, 靶向治疗, Lung neoplasms, Adenocarcinoma, Squamous cell carcinoma, Oncogenes, Targeted therapy

## Abstract

腺癌和鳞癌是非小细胞肺癌（non-small cell lung cancer, NSCLC）最常见的两种病理类型。一些诱发和维持恶性肿瘤的分子改变被称为驱动基因。随着多重基因分型和高通量基因组分析等下一代（next-generation sequencing, NGS）测序技术的推广运用，使得从微小的肿瘤活检标本中检测病患者的癌症基因组成为可能，基于基因特征的临床研究也相继开展，进一步推动对NSCLC的分子分型。肺腺癌中约60%的驱动基因被确定，肺鳞癌驱动基因的检出率也在逐步提高，其中表皮生长因子受体（epidermal growth factor receptor, EGFR）、间变性淋巴瘤激酶（anaplastic lymphoma kinase, ALK）、成纤维细胞生长因子受体1（fibroblast growth factor receptor 1, FGFR1）、磷脂酰肌醇3激酶催化亚单位A（phosphatidylinositol 3-kinase catalytic subunit alpha, PIK3CA）等起着重要作用，目前临床上有效的驱动基因靶向治疗主要是针对EGFR、ALK等。本文将对NSCLC驱动基因的意义及相关研究进行综述。

## Abstract (en)

Adenocarcinoma and squamous cell carcinoma are the most common histological types of non-small cell lung cancer (NSCLC). Several molecular alterations have been defined as "driver oncogenes" responsible for both the initiation and maintenance of the malignancy. With next-generation sequencing (NGS) which having multiple and high-throughput genotyping is wildly used and promoted, make the detection of patients gene composition from a tiny tumor biopsy specimens become possible, initiate the clinical studies based on the genetic characteristics, and promote the progress of molecular typing in NSCLC. So far, about 60% of lung adenocarcinoma has been found harbouring driver oncogenes, the rate of lung squamous cell carcinoma driven genes detection has gradually improved, in which epidermal growth factor receptor (EGFR), anaplastic lymphoma kinase (ALK), fibroblast growth factor receptor 1 (FGFR1), phosphatidylinositol 3-kinase catalytic subunit alpha (PIK3CA) and so on plays important roles. The currently effective targeted therapies is mainly used against EGFR, ALK, *etc*. In this review, we will report the mainly advances on some latest driver mutations of NSCLC.

肺癌是目前癌症死亡的主要原因^[1]^。非小细胞肺癌（non-small cell lung cancer, NSCLC）约占肺癌的85%^[1]^。腺癌（adenocarcinoma, AC）、鳞状细胞癌（squamous cell carcinoma, SCC）是NSCLC最常见的两种病理类型，其中AC和SCC约占70%^[2]^。多数NSCLC患者在确诊时已属晚期^[2]^，含铂联合化疗可以延长患者的生存期，但是晚期NSCLC患者的预后仍然极差，5年生存率小于15%^[3]^。

随着基因分析和分子诊断技术的不断发展，比如下一代（next-generation sequencing, NGS）测序技术^[4]^推广运用，使得从微小的肿瘤活检标本中检测患者的癌症基因组成为可能，基于基因特征的肺癌临床研究也相继开展。表皮生长因子受体酪氨酸激酶抑制剂（epidermal growth factor receptor-tyrosine kinase inhibitors, EGFR-TKIs）治疗*EGFR*敏感突变肺癌取得良好的疗效^[5, 6]^。棘皮动物微管样蛋白4-间变淋巴瘤激酶（echinoderm microtubule associated protin like 4-anaplastic lymphoma kinase, *EML4-ALK*）融合基因抑制剂克唑替尼（crizotinib）在特定分子分型的肺癌中也显示出神奇的抗肿瘤活性^[7]^。

由此可见，仅仅依靠组织病理类型制定治疗方案和选择治疗药物已无法充分实现对肺癌患者的个体化治疗。在制定肺癌的治疗策略时，除考虑患者的性别、年龄、体能状况、病理类型、肿瘤TNM分期以外，更应重视肿瘤的基因特征。在过去的十年间，NSCLC驱动基因的研究取得了明显的进步，尤其是肺腺癌，约60%的驱动基因被确定，肺鳞癌驱动基因的检出率也在逐步提高。本文就近年来NSCLC驱动基因的相关研究作一综述。

## 1 肺腺癌的驱动基因

EGFR酪氨酸激酶突变体的发现开启了NSCLC靶向治疗之门，它是肺腺癌常见的驱动基因。从非选择到选择特定患者、从选择病理类型到检测基因突变，对EGFR-TKIs的研究经历了去伪存真、由表及里地不断探索。EGFR-TKIs治疗*EGFR*敏感突变患者的成功，奠定和推动了肺癌的个体化治疗。近期针对*ALK*融合基因NSCLC靶向治疗的再次成功极大激发了对肺癌驱动基因的研究^[8]^。

肺腺癌中可发挥功能性驱动基因的突变率约60%，其中*KRAS*（kirsten-rous avian sarcoma）、*EGFR*突变和*EML4-ALK*融合是最常见的驱动基因，约占35%-40%^[9]^。2011年美国临床肿瘤学会（American Society of Cliniacal Oncology, ASCO）会议上报道了美国国立癌症研究所肺癌突变联盟（LCMC）的一项研究^[10]^，对1, 007例进展期（Ⅲb期/Ⅳ期）肺腺癌患者检测10种已知的癌症驱动基因，并按不同驱动基因分组对其生存进行记录。此项研究在2013世界肺癌大会（World Conference on Lung Cancer, WCLC）上进行了数据更新，结果显示：在63%的患者中发现驱动基因，发生率前3位依次是*KRAS*（25%）、*EGFR*（敏感型，15%）和*ALK*（8%），其他常见的驱动基因分别是*EGFR*（其他型）、双基因变异、*HER2*（human epidermal growth factor receptor-2）、*BRAF*、*PIK3CA*、*MET*、*NRAS*，其发生率均小于5%；研究还发现有驱动基因且接受靶向治疗者的中位生存期较未接受靶向治疗者明显延长（3.5年*vs* 2.4年），也优于驱动基因未知者（2.1年）；在5种最常见的驱动基因中，*ALK*、*EGFR*（敏感型）和*EGFR*（其他型）的中位生存期较*KRAS*和双基因变异者明显延长（分别是4.3年、4年、3.3年*vs* 2.4年和2年）。2012年ASCO会议上报道一项名为PIONEER的研究结果^[11]^，对入组的来自亚洲7个国家和地区的1, 482例Ⅲb期/Ⅳ期肺腺癌组织标本进行*EGFR*突变检测。*EGFR*基因总突变率为51.4%；女性突变率为61.0%，男性为44.0%；不吸烟者突变率为60.7%，重度吸烟者为31.4%；各国或地区*EGFR*突变率分别为：越南62.2%、台湾62.1%、泰国53.8%、菲律宾52.3%、中国50.2%、香港47.2%、印度22.2%；其中人种和吸烟状况是影响突变最重要的因素。2013年ASCO会议上报道了一项法国的样本量为10, 000例的肺癌生物标志物（biomarker, BM）项目研究^[12]^，前瞻性地筛查了*EGFR*、*ALK*、*HER2*、*KRAS*、*BRAF*、*PIK3CA*等6种生物标志物的突变，在可评价的9, 911例患者中腺癌占76.1%，46%的肺癌样本中检测到已知靶点变异，其中*KRAS*突变为27%、*EGFR*活化突变为9.5%、*ALK*基因融合为3.7%、*PIK3CA*突变为2.6%、*HER2*突变为0.9%、*BRAF*突变为1.7%，*EGFR*其他突变为0.8%，其分子分型结果指导了57%患者的实际治疗决策。

肺癌腺除上述驱动基因外，目前尚有*ROS1*、*KIF5B-RET*融合基因等研究的相关报道。1982年*ROS1*在UR1鸟肉毒病毒中被确定为具有独特致癌作用的病毒原癌基因^[13]^，*ROS1*基因重排与*ALK*基因重排在2007年同时被发现于NSCLC^[14]^，*ROS1*基因重排阳性具有与ALK相似的临床特征，即年轻、从不吸烟、且为高恶性度肺腺癌患者^[15]^，但其发生率远低于ALK重排，约为1%-2%，而在EGFR/KRAS/ALK均阴性的人群中的发生率可达5.7%^[16]^，ROS1重排阳性患者预后较阴性者差^[15]^。因为与ALK重排阳性患者具有相似性的临床特征，因此有研究者采用crizotinib治疗*ROS1*基因重排NSCLC患者。2013年ASCO年会上报道了较大样本的Ⅰ期临床试验，包括31例ROS1阳性晚期NSCLC患者，在治疗8周和16周时患者的疾病控制率分别达到76%和60%，治疗总缓解率为56%，包括2例完全缓解，12例部分缓解和8例疾病稳定，6个月无进展生存率达到71%，表明crizotinib对ROS1重排阳性NSCLC患者有明显治疗效果^[15, 17]^。*KIF5B-RET*融合基因是*EGFR*、*KRAS*、*EML4-ALK*等驱动基因之外另一个重要的EGFR-TKIs作用靶点，该融合基因在不吸烟或少量吸烟、腺癌、无*EGFR*及*KRAS*突变、无*EML4-ALK*融合基因的NSCLC患者中发生率较高。在欧美的大样本研究^[18]^中，不吸烟的白人NSCLC中*KIF5B-RET*基因融合频率约为1.8%（12/667例），而在无EGFR、KRAS、HER2、ALK、ROS1变异的肺癌中，*RET*基因的融合频率高达6.3%（10/159例）。在日本的大样本研究^[19]^中，在1, 529例肺癌样本中，*ALK*、*ROS1*、*KIF5B*融合基因在所有肺癌和肺腺癌中的出现频率分别为3.0%（腺癌3.9%）、0.9%（腺癌1.2%）、0.9%（腺癌1.2%）。*KIF5B-RET*融合基因NSCLC患者可能对舒尼替尼、索拉非尼和凡德他尼敏感，需要前瞻性临床研究进行验证。

## 2 肺鳞癌的驱动基因

尽管SCC是目前肺癌第二常见的病理类型，但是对驱动基因的研究大多集中在腺癌、从未吸烟或轻度吸烟者，因此对肺鳞癌基因改变的认识明显滞后于腺癌。

Kan等^[20]^在确定的1, 507个候选基因中有967个基因发现了2, 500个体细胞突变事件，肺鳞癌中突变的癌基因明显不同于腺癌，鳞癌中*TP53*、*GRM8*、*BAI3*、*ERBB4*、*RUNX1T1*、*KEAP1*、*FBXW7*、*KRAS*等基因突变比较常见。在Heist等^[21]^的综述中，肺鳞癌常见的驱动基因及发生频率分别为*FGFR1*扩增（20%）、*SOX*扩增（20%）、*PIK3CA*扩增（20%）、*MDM2*扩增（10%）、*PDGFRA*扩增（8%-10%）、*MET*扩增（6%）、*P53*突变（65%）、*NRF2*突变（10%-15%）、*PTEN*突变（10.2%）、*EPHA2*突变（7%）、*AKT*突变（5%）。而Drilon等^[22]^报道的肺鳞癌中常见驱动基因及发生频率分别为*FGFR1*扩增（20%）、*PTEN*突变（10%）、*AKT1*突变（6%）、*DDR2*突变（4%）、*PIK3CA*突变（4%），另有56%为基因状态未知。韩国的研究^[23]^发现104例肺鳞癌标本中*P53*、*PTEN*、*PIK3CA*等7种驱动基因突变频率与北美肺鳞癌标本相似。

SOX2是一种转录因子并且对胚胎和神经干细胞严格调控，在肺上皮细胞的发展中发挥关键作用。RNA干扰敲除SOX2可减少细胞增殖，抑制SOX2比PIK3CA和TP63对细胞生长的影响更大^[24]^。SOX2过表达可导致细胞迁徙、锚定和独立生长，而SOX2基因敲除则可影响细胞生长^[25]^。SOX2过度表达可导致小鼠模型广泛增生和癌变，有证据^[25]^表明，SOX2扩增可能只是一个“启动事件”，需要下游信号通路进一步改变才能驱动癌变。目前没有SOX2抑制剂在临床试验中使用。FGFR1是FGFR家族的受体酪氨酸激酶的成员，通过PI3K/AKT、RAS/MAPK激活下游信号传导，促使肿瘤生长、迁徙和血管生成等。在肺鳞癌的FGFR1扩增比腺癌常见，约20%肺鳞癌具有FGFR1扩增，在细胞株和小鼠模型抑制FGFR1显示可抑制细胞生长和诱导凋亡^[26]^。*FGFR1*基因突变非常罕见^[22]^。FGFR抑制剂正在研发中，多为多靶点TKIs，可针对除FGFR1外的其他靶点。针对FGFR1扩增肺鳞癌的多个临床研究正在进行中。PI3K-AKT通路在许多癌症的存活和增殖起着核心作用，PIK3CA扩增及突变在肺癌均已确定，扩增可导致PI3K通路细胞信号激活。有多个PI3K抑制剂正在开发中，特异性地针对PI3K/MTOR、panPI3K等。临床前数据^[22]^表明，PIK3CA激活突变对PI3K通路抑制剂最为敏感，PI3K抑制剂与其他信号通路抑制剂联合应用的研究正在积极进行中。体细胞*PTEN*基因缺失、突变和PTEN失活，如甲基化或小分子RNA沉默失活，可出现在多种癌症。无论是腺癌和鳞状细胞，PTEN表达下降或不表达发生率均高达70%，肺鳞癌*PTEN*基因突变高于腺癌（10.2% *vs* 1.7%）。虽然缺乏确切的数据，但是*PTEN*基因丢失肺癌可能对PI3K途径抑制剂更敏感，PI3K抑制剂治疗PTEN丢失患者的临床试验正在进行中^[23]^。由于缺乏针对肺鳞癌驱动基因有效的靶向治疗药物，且多数尚在临床研究初期，多为Ⅰ期、Ⅱ期临床研究，所以目前肺鳞癌分子分型并未给临床带来更多的获益。

## 3 NSCLC驱动基因的靶向治疗

虽然NSCLC驱动基因检测取得了可喜的进展，肺腺癌中约60%的驱动基因被确定，肺鳞癌驱动基因的检出率也在40%-50%，但是针对NSCLC驱动基因的靶向治疗药物仍然非常有限，而且主要是针对肺腺癌的驱动基因，比如EGFR-TKIs、ALK-TKIs等。

针对EGFR的治疗是目前NSCLC中应用最广、疗效最确切的驱动基因靶向治疗，尤其是EGFR-TKIs。吉非替尼（Gefitinib）是首个口服的EGFR-TKI，在IDEAL Ⅰ和IDEAL Ⅱ两项随机双盲Ⅱ期临床研究中，化疗耐药NSCLC患者的有效率达到8.8%-19.0%，症状缓解率可达35%-43%，显示该药对NSCLC患者二线或三线治疗确切的疗效^[27]^。在IPASS研究^[5]^中，EGFR-TKIs作为晚期NSCLC的一线治疗与标准一线化疗比较，结果显示：在腺癌、不吸烟或已经戒烟的轻度吸烟者的亚裔晚期NSCLC患者中，口服Gefitinib相对于紫杉醇联合卡铂化疗方案，具有无疾病进展生存期（progression free survival, PFS）方面的优势；亚组分析显示，*EGFR*突变阳性的患者使用Gefitinib，其PFS明显长于化疗患者，*EGFR*突变状态不明的患者使用吉非替尼其PFS更长，与总体人群的结果一致。厄洛替尼是另一个口服EGFR-TKI，在BR.21研究^[28]^中，采用厄洛替尼与最佳支持治疗既往化疗失败的晚期NSCLC患者，与安慰剂组相比，厄洛替尼的有效率更高（8.9% *vs* < 1%）、PFS更长（2.2个月*vs* 1.8个月）、总生存期延长（6.7个月*vs* 4.7个月）、1年生存率更高（31% *vs* 22%）。进一步确立了EGFR-TKIs在化疗耐药NSCLC患者治疗中的地位。在SATURN研究中，厄洛替尼维持治疗取得了明显的阳性结果，一线化疗达到疾病稳定（stable disease, SD）的患者无论临床特征或分子学特征都可以延长总生存期。埃克替尼是我国第一个拥有自主知识产权的小分子靶向抗肿瘤药，是一种强效、高选择性的口服酪氨酸酶抑制剂。在ICOGEN Ⅲ期临床研究^[29]^中，埃克替尼总体疗效不逊于吉非替尼，无进展生存期4.6个月*vs* 3.4个月；皮疹、腹泻等发生率与吉非替尼相似；埃克替尼组的药物相关不良反应事件更少（61% *vs* 70%, *P*=0.046），尤其是药物相关性腹泻（19% *vs* 28%, *P*=0.033）。单克隆抗体阻断配体与EGFR结合是一种针对*EGFR*驱动基因的治疗策略。在FLEX研究^[30]^中，西妥昔单抗联合长春瑞滨/顺铂（NP方案）一线治疗EGFR表达阳性的晚期NSCLC患者，总生存期明显长于化疗组（11.3个月*vs* 10.1个月）。多西紫杉醇单药联合西妥昔单抗治疗一线化疗失败或3个月内复发的EGFR表达阳性NSCLC患者，ORR为20%，SD为36.4%，中位生存期为7.5个月，1年生存率达35%^[31]^。显示出EGFR单克隆抗体在NSCLC的一线和二线治疗中的良好前景。

目前针对*ALK*融合基因NSCLC的研究比EGFR更为热门。ASCO会议连续四年均有报告涉及克唑替尼。克唑替尼Ⅰ期临床试验^[32]^是驱动基因指导的研究，入组标准为ALK阳性的NSCLC患者，总缓解率（objective response rate, ORR）为57%，治疗8周时的临床获益率为87%，6个月的PFS率为72%。2011年ASCO会议上报道的Ⅱ期临床研究（PROFILE 1005）^[33]^采用克唑替尼治疗晚期ALK阳性NSCLC患者，133例可评价疗效患者的ORR为51.1%，6周和12周的疾病控制率（disease control rate, DCR）分别为85%和73.3%。2012年欧洲内科肿瘤学会（European Society for Medical Oncology, ESMO）更新了PROFILE 1005的研究结果，259例可评价疗效患者的ORR为60%，中位PFS为8个月，且安全性良好^[34]^。其结果支持了Ⅰ期临床研究中显示的有效性和安全性。2012年ESMO报道的PROFILE1007研究^[35]^，将318例一线化疗失败的NSCLC患者随机分为克唑替尼组和培美曲塞或多西他赛标准二线化疗药物组，ORR分别为65.7%、29.3%和6.9%，PFS分别为7.7个月、4.2个月和2.6个月，克唑替尼治疗组明显优于化疗组。2014年ASCO会议上报道了crizotinib用于一线治疗的PROFILE1014研究^[36]^，与标准化疗方案培美曲塞+顺铂或培美曲塞+卡铂进行头对头比较，343例ALK阳性的非鳞初治NSCLC患者，按照1:1的比例随机接受克唑替尼（172例）或标准化疗（171例），结果显示克唑替尼明显优于化疗，ORR分别为74%和45%，PFS分别为10.9个月和7.0个月，68%的患者仍在随访中，OS结果目前尚未显示出明显的改善。由此可见，crizotinib无论是一线或是二三线治疗*ALK*融合基因阳性的NSCLC患者均显示了良好的抗肿瘤活性，并且安全、方便。目前以中国患者为主的PROFILE1029等研究也正在进行中。

ROS1与ALK同属胰岛素样受体酪氨酸激酶超家族成员，二者氨基酸序列上具有近49%的相似性，且都对crizotinib敏感。2012年ASCO会议上首次报道了crizotinib治疗具有*ROS1*融合基因NSCLC患者的Ⅰ期临床研究^[37]^结果，在13例可评价疗效的患者中，ORR为54%，8周DCR为85%。2012年ESMO上该研究进行了数据更新，在20例可评价疗效的患者中，ORR为50%，8周DCR为70%。研究结果^[38]^提示crizotinib对具有*ROS1*融合基因这一新驱动基因的NSCLC患者的疗效明显。

肺SCC驱动基因进入临床研究的并不多见。使用FGFR抑制剂-PD173074治疗移植FGFR1扩增肺癌细胞系的小鼠模型，可以观察到肿瘤消退^[39]^。1例FGFR1扩增的肺癌患者经过治疗，8周时靶病灶缩小33%，12周时病灶仍然维持缩小^[40]^。2014年ASCO会议上报道一项FGFR1-TKI（AZD4547, BGJ398）的Ⅰ期临床研究^[41]^，入组的21例FGFR1扩增的肺SCC患者接受BGJ398 125 mg/d治疗，在17例可评价疗效的患者中，4例患者部分缓解（2例在数据截止日后达到部分缓解），其中2例部分缓解疗效维持达8个月和3个月，另有3例患者疾病稳定，不良反应主要为高磷血症、口腔炎、脱发、食欲下降和疲劳等。肺SCC中*PI3KCA*基因的扩增远较突变常见，在Ignacio等^[42]^的一项早期研究中，采用PI3K/Akt/mTOR轴抑制剂治疗*PI 3KCA*基因突变NSCLC患者，23%（18/78）的患者获得6个月以上的疾病稳定或完全缓解及部分缓解。两项代号分别为NCT00974584和NCT0075684采用口服PI3K激酶抑制剂-GDC-0941治疗非选择人群的研究正在进行中。由此可见，FGFR1、PI3KCA等驱动基因有望成为肺鳞癌新的治疗靶点。

## 4 小结与展望

多重基因分型和高通量基因组分析等NGS技术可以更快更准确地确定基因分型，肺腺癌中约60%的驱动基因被确定，肺鳞癌驱动基因的检出率也在逐步提高。目前NSCLC正已逐渐由标准化、“一刀切”的治疗模式向基于基因状况的个性化治疗转变。EGFR-TKIs、ALK-TKIs在特定驱动基因的NSCLC患者中获得了奇迹般的疗效，使得肺腺癌在个体化治疗中初露锋芒。目前基因分型并未给肺鳞癌带来明确的临床获益，针对肺鳞癌驱动基因的靶向治疗研究正在进行中，多为Ⅰ期、Ⅱ期临床研究，期待有更多的研究开展并取得突破。NGS技术使得基因分型与遗传学检测更便捷、更准确，但仍需进一步开发，以适用于在有限组织标本情况下能同时满足组织病理检查及分子病理学检测要求。总之，NSCLC基因分型与相关靶向治疗已取得初步进展，但仍只是初露端倪，需要进一步持续、深入地研究。
